# Identifying patient-level data risks in trusted research environments: Worked examples with synthetic data

**DOI:** 10.1177/20552076261440981

**Published:** 2026-06-23

**Authors:** M. Baragilly, A. Topham, S. Gallier, E. Sapey

**Affiliations:** 1 Department of Inflammation and Ageing, College of Medicine and Health, 1724 University of Birmingham, Birmingham, UK; 2 Department of Mathematics, Insurance and Applied Statistics, Capital University, Cairo, Egypt; 3 PIONEER Health Data Research Hub for Acute Care, 1732 University Hospitals Birmingham NHS Foundation Trust, Birmingham, UK; 4 Research & Innovation Department, 1732 University Hospitals Birmingham NHS Foundation Trust, Birmingham, UK; 5 NIHR West Midlands Applied Research Centre, 1724 University of Birmingham, Birmingham UK; 6 NIHR Midlands Patient Safety Research Collaboration, 1724 University of Birmingham, Birmingham, UK; 7 University of Birmingham, School of Medical Sciences, Edgbaston, Birmingham, UK; 8 NIHR Birmingham Biomedical Research Centre, 1732 University Hospitals Birmingham NHS Foundation Trust, Birmingham, UK

**Keywords:** trusted research environments (TREs), synthetic data, data egress, privacy-preserving, data security

## Abstract

**Objective:**

This study examined and illustrated real-world risks of unintended patient-level data egress from Trusted Research Environments (TREs) and Secure Data Environments (SDEs), using synthetic data to recreate cases encountered in PIONEER, the HDR UK Hub in Acute Care.

**Methods:**

Synthetic datasets with demographics and NEWS2 vital signs were created using SciPy and NumPy for two fictitious populations. These datasets were transformed for machine-learning and embedded into various formats to simulate potential egress scenarios. Three worked examples include binary serialisation of data, binary serialisation of complex objects, and plain text mark-up reports.

**Results:**

Initial screening of exported files included checking reported sizes. While absolute size alone cannot confirm patient-level data, unusually large files can signal the need for closer inspection. In several cases, this prompted manual review that uncovered sensitive information. File size is therefore a useful signal within a layered egress checking process, not a diagnostic measure. Standard tools like Python or R do not warn of hidden data, reinforcing the need for explicit egress policies and independent verification. Converting binary formats only works for recognized code libraries and requires ongoing maintenance. Manual inspection alongside automation remains essential to identify and remove embedded data.

**Conclusion:**

These cases highlight the complexities in identifying and preventing identifiable data egress from TREs. Key insights include clear guidance for researchers, the limitations of binary serialisation for egress due to security vulnerabilities, and the importance of plain-text data exports for ease of verification.

## Introduction

Data security in health and social care research has increasingly focused on the use of TREs to protect sensitive information, yet the risks of data egress from these environments remain underexplored. To assess the existing body of evidence, we conducted a PubMed search on September 03, 2024, using the terms “Trusted Research Environments” OR “Secure data environments” AND “disclosure” OR “Data egress” OR “Data breach” AND “health data security,” without language restrictions and limited to publications from 2014 to the present. This search yielded 287 results, only 1 publication directly addressed the risks of data egress from TREs, and even fewer provided empirical insights or worked examples relevant to health data and the risks of disclosure.

Our review identified that most literature focused on general privacy frameworks or the theoretical risks highlighting a notable gap in practical, evidence-based resources. No studies were found that demonstrated specific methods by which data could be unintentionally egressed via code or machine-learning outputs within a TRE. Notably, no systematic reviews or meta-analyses exist to quantify the risk of data egress from TREs.

This study provides empirical evidence through worked examples, demonstrating specific scenarios where individual-level patient data could be unintentionally egressed from TREs within machine-learning and data science outputs. Using synthetic datasets, we simulated three scenarios involving binary serialisation of data, complex objects, and plain text mark-up reports. Current automated egress screening processes may overlook embedded data within these formats, underscoring the limitations of existing tools in managing egress risks in TREs. This study provides practical insights into how such risks manifest in real-world settings, offering specific guidance for data controllers on mitigating these risks.

This will have implications for policy and practice, especially within the context of the Department for Health and Social Care’s ongoing transition to a model where health and social care data are accessed exclusively through SDEs. Our work demonstrates the current need for manual egress review processes alongside the development of automated tools. For future research, these findings prompt a need to develop more advanced, adaptable egress screening systems capable of detecting hidden data within complex code outputs. This study provides a foundation for further exploration into safeguarding data within TREs, helping align practice with the growing demand for secure, responsible data access in healthcare research.

Health and social care data are increasingly expected to be accessed for research within Trusted Research Environments (TREs), otherwise referred to as Secure Data Environments (SDEs).^1,2^ TREs enable researchers to access data for analysis, overseen through physical systems, processes and contractual arrangements such as data licensing agreements. Data remains within the TREs, eliminating the need to egress patient-level data to external researcher-controlled environments. The TRE framework aligns with the “five-safes” framework for privacy and providing greater reassurances to patients, the public and data controllers about the safety and security of accessing data for research.^3,4^

Several initiatives have established TREs to support specific research purposes. Examples include the Health Data Research UK (HDR UK) founded hub, PIONEER focusing on Acute Care,^5^ the UK Longitudinal Linkage Collaboration (UK LLC)^6^ focusing on COVID-19 records, and the Safe Haven Artificial Platform (SHAIP)^7^ which provides a secure environment for AI algorithm development using anonymised National Health Service (NHS) imaging and reports. At a national level, the Secure Anonymised Information Linkage (SAIL) Databank^8^ serves as the TRE for Welsh NHS and social care data.

In addition to England and Wales, Scotland operates the Scottish Safe Haven Network, a national framework of accredited safe havens supporting research while protecting patient confidentiality.^9^ Northern Ireland provides similar secure access through the Honest Broker Service, which enables the use of de-identified health and social care data for approved research and service evaluation projects.^10^ These initiatives complement England’s regional SDE programme and the Secure Anonymised Information Linkage (SAIL) Databank in Wales, together forming a UK-wide infrastructure for privacy-preserving data research.

Recently, NHS England (NHSE) funded 12 regional SDEs to facilitate access to linked regional health data.^11^ The policy surrounding this initiative emphasises a shift towards a model where all NHS data is accessed through NHS-controlled SDEs. The TRE model operates under the assumption that the risk of egressing potentially identifiable patient data (either by mistake or maleficence) is low, given egress permissions are tightly controlled. However, the management of SDEs has become increasingly complex as health datasets grow larger and more diverse, and as researchers apply advanced analytics and machine-learning models that can inadvertently embed or reconstruct identifiable patient information. These trends demand more sophisticated governance and technical safeguards to prevent unintended data egress. Although this study draws its real-world examples from the NHS, the challenges of preventing unintended patient-level data egress and ensuring secure research environments are common to health-data TREs worldwide. The lessons and guidelines presented here are therefore applicable to a wide range of international TRE settings.

There is little published guidance on managing data egress from TREs, which remains a key risk for potential data egress.^12^ While projects such as GRAIMATTER project have identified theoretical risks of machine-learning models exposing sensitive health information during output,^13,14^ the sector lacks worked, real-world examples that demonstrate these risks in actionable terms.

To situate these challenges within a wider governance context, egress checking can be understood as a socio-technical process that relies on multiple complementary layers of oversight. This aligns with established “defence-in-depth” models, in which risk is mitigated through several imperfect but overlapping safeguards rather than any single mechanism.^15,16^ In TREs, these safeguards fall along an *automation–manual inspection spectrum*. Automated and semi-automated checks, such as file size thresholds, metadata inspection, and format validation; provide rapid signals of potential issues but cannot reliably determine whether sensitive information is present.^17,18^ Cybersecurity and data governance principles reinforce that human judgment is essential for identifying contextual risks that automated tools cannot detect.^19^ Current TRE guidance similarly emphasises layered verification, tool maintenance, and expert review as core components of safe data release.^20^

The aim of this work was to support TRE data controllers to identify potential cases where data egress could occur. We provide worked examples of where individual patient-level data could be egressed from TREs within analytical outputs, without this being obviously apparent to TRE data controllers. The chosen examples are based on cases where data egress was prevented and individual level patient data removed prior to outputs being shared. For this study, examples have been replicated using synthetic data. Sample files are provided for reference in the online supplementary material, illustrating how the data concerned might look within analytical code or algorithms. The worked examples presented here are intended to demonstrate plausible technical mechanisms and governance challenges associated with data egress rather than to estimate prevalence, frequency, or risk magnitude across all Trusted Research Environments.

## Methods

This methodological demonstration study^21^ was conducted between January and October 2024 within the PIONEER Trusted Research Environment (TRE) at University Hospitals Birmingham NHS Foundation Trust, United Kingdom. The study aimed to illustrate mechanisms through which unintended patient-level data egress could occur using synthetic worked examples based on real-world operational scenarios.

Selection criteria for worked examples included: (1) representation of realistic data egress scenarios encountered during TRE operations; (2) demonstration of different technical mechanisms through which patient-level data could be embedded within outputs; and (3) coverage of varying levels of technical complexity and detectability. Scenarios were excluded if they did not involve plausible real-world analytical workflows or did not illustrate mechanisms relevant to TRE governance.

### Synthetic data generation

All examples used the same dataset, namely a set of demographics and NEWS2 physiological measurements for two fictitious sub-populations of one hundred patients each, with respiratory rate, respiration type (oxygen or room air), systolic blood pressure, pulse rate, body temperature, consciousness level, generated at random by drawing from a reasonably weighted distribution of values, and ethnicity, age and sex sampled randomly across uniform distributions drawn from ages between 18 and 95, sex values of either “male” or “female” and ethnicity values from the Office for National Statistics (Online Supplemental File S01a). Data were generated using the random numerical distributions in the SciPy package in the scipy. stats namespace, and categorical variables were drawn using NumPy’s random. choice method, using the parameters in Table 1.^22,23^ Code to generate the sample data is in Online Supplementary File S01, including seed values for all pseudorandom number function calls, and the dataset itself is in Online Supplemental File S02a. Ethnicity values are from the Office for National Statistics.^24^

**Table 1.** Parameters used to generate random data for file samples presented herein.

Vital sign	Distribution	Population	Parameter	Value
Blood O_2_ Saturation	Beta	1	alpha	5
			beta	0·25
		2	alpha	3
			beta	0·25
Systolic Blood Pressure	Normal	1	mean	128
			standard deviation	5
		2	mean	105
			standard deviation	5
Pulse Rate	Normal	1	mean	70
			standard deviation	15
		2	mean	70
			standard deviation	15
Temperature	Normal	1	mean	37
			standard deviation	0·5
		2	mean	38
			standard deviation	0·5
Respiratory Rate	Normal	1	mean	16
			standard deviation	2
		2	mean	19
			standard deviation	2
Supplementary Oxygen	Random Choice	1	oxygen probability	0·1
			room-air probability	0·9
		2	oxygen probability	0·2
			room-air probability	0·8
Consciousness (ACVPU)	Random Choice	1	alert probability	0·85
			CVPU probability	0·15
		2	alert probability	0·78
			CVPU probability	0·22
Sex	Random Choice	1	male probability	0·57
			female probability	0·43
		2	male probability	0·43
			female probability	0·57
Ethnicity	Random Choice	equal probabilities across all values		
Age	Random Choice	equal probabilities across all values		

Pulse rate was selected to be an uninformative variable, and so parameters were the same for both populations. Age values were all whole integers ranging from 18 to 95. Ethnic groups were selected by equal probability, samples from the Office for National Statistics high-level ethnic categories for the 2021 census.

To generate further example files for the case studies, the randomly generated data in Online Supplemental File S02a was first feature-transformed into a dataset compatible with Machine-learning methods (Online Supplemental File S02c) using code as shown in Online Supplemental File S02b), with one-hot encoded columns for ethnicity, sex, consciousness and air supply (“breathing”), numerical encoding of population and the original, continuous variables left unchanged (age, pulse, blood pressure, temperature and respiratory rate), and finally example files were generated using models trained in Supplemental File S02d.

To generate the code with extractable, embedded patient-level data 100 randomly generated sets of synthetic patient data were developed containing demographics, outward code (first half of postcode), and some inferred sensitive diagnoses along with times of admission and discharge. This was then serialised to JSON and a copy of the Jupyter Notebook (.ipynb) used to generate the random data above was opened in a plain text editor and the serialised data manually inserted at line 173 (.ipynb files are themselves JSON formatted data). Opening the notebook in in Visual Studio Code (VS Code) confirms that the notebook is rendered as expected and the inserted patient data is not visible when rendered as such, i.e. is hidden from view when examining the file with one of its intended applications. All development work was performed in VS Code version 1·89·1 with the Python extension version v2024·6·0, Jupyter extension version v2024·4·0 and Pylance version v2024·5·1, using Python version 3·10·5.

The risk caused by the analytical techniques described in the case studies was rated from low to high depending on the visibility of the patient-level data, with low risk being defined as anything where sensitive data are explicitly identifiable and easily inspected, and high risk being defined as instances where sensitive data is concealed in unusual and/or technically demanding file formats. Three representative egress scenarios were developed using the synthetic dataset to illustrate different pathways through which patient-level data might be unintentionally exported from a TRE:

1. Binary Serialisation of Data: an AI performance file created using Python “pickle” that inadvertently includes the training data matrix.
2. Binary Serialisation of Complex Objects: a multilayer object containing a Support Vector Machine (SVM) model where support vectors preserve elements of the original training data.
3. Plain-Text Mark-up Reports: an HTML report generated by an R library embedding spreadsheet data as Base-64-encoded Excel files and unsuppressed small-count aggregates.

These three scenarios were selected to represent increasing technical complexity and different common file formats encountered in TREs.

### Statistical analysis

No inferential statistical testing was performed, as the study aimed to demonstrate technical mechanisms rather than estimate population effects. Statistical methods were limited to synthetic data generation and preprocessing. Continuous variables were simulated using weighted probability distributions implemented via SciPy, and categorical variables were generated using NumPy random sampling functions. Feature-transformation was performed to create machine-learning compatible datasets for illustrative purposes.

## Results

### Case one: Binary serialisation of data

**Figure 1.**
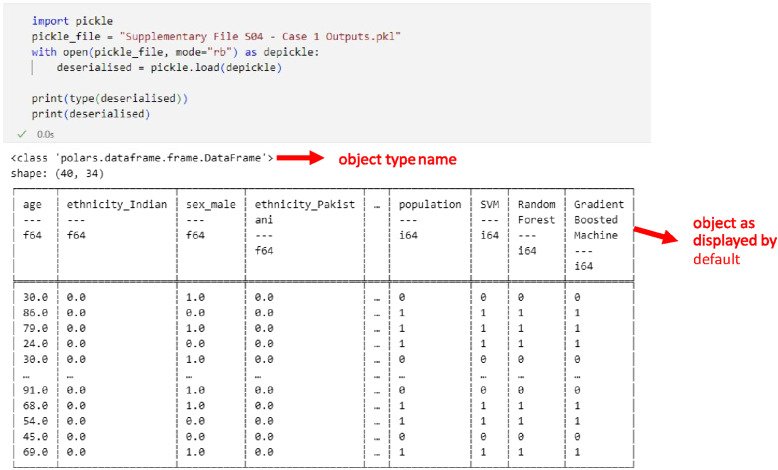
Case one as viewed in VS code. Screenshot taken from VS code of case one example output file, showing the object type name and the contents of the object highlighted in red. In this instance, the default display rendering of the object presented the data in a clear and very readable way, and the object type immediately reveals the nature of the object if not the content.

This case involves a request to egress performance data from an artificial intelligence generated algorithm trained on patient-level data. The use of Python’s binary object serialisation (“pickle”) to save the performance data matrices serialises other objects from memory to disk, including the matrices used to train the models. This data matrix includes patient-level data. Online Supplemental File S04 contains a worked example of a binary-serialised dataset which includes patient-level data. Figure 1 provides a series of marked screenshots demonstrating the embedded synthetic data within the code.

In this instance the data were easily identifiable by the object type in memory, although this is easily circumvented by, for instance, namespace confusion attacks, a type of supply-chain attack which seeks to replace officially recognised public packages with other, undesirable packages using the same names to confuse, and hopefully bypass security checks. This object (a Data Frame from the Polars python package) has a default display format that is easy to interpret.

This case highlights two challenges: first, even when researchers are aware of what constitutes permissible egress data, tools like object serialisation or disorganised file management can easily result in including row-level patient data among outputs. Second, binary object serialisation complicates output verification. Even plain-text serialisation like JSON or XML requires some expertise to interpret.

In this instance, the “pickled” files were read back into Python, which throws informative exceptions when dependencies are missing, allowing researchers to open them for inspection. The object’s type indicated its likely contents, but verifying its safety still required manual inspection, as a namespace confusion attack could circumvent this process.

### Learning

File size alone did not provide a clear indication of the presence of patient-level data, as serialised binary files can vary in size. For example, a .csv table of aggregate statistics with tens of rows of data and approximately ten columns (as might be expected of a table of aggregate summaries) would be on the order of a few kilobytes in size, whilst the same format with tens of columns and thousands of rows of data (such as one might expect of patient-level data) would likely be greater than a Megabyte in size. Additionally, the Polars Python package did not generate any explicit warnings or alerts to indicate that patient-level data might be embedded within the serialised object. The lack of an in-built warning mechanism or automated detection capability emphasises a gap in current methodologies for verifying serialised objects, especially when faced with sophisticated techniques such as namespace confusion attacks.

Robust monitoring strategies might include prohibiting the export of binary-serialised files where possible, to prevent the inadvertent exfiltration of patient data. While partial automation is feasible by developing tools that convert commonly used binary formats into more interpretable forms like .csv, this approach has limitations. It is only effective for code libraries that the automation explicitly recognises and requires continual maintenance to keep pace with updates to the target libraries. Thus, a combination of improved automated solutions, package-specific warnings, and manual review is necessary to mitigate the risk of data egress.

### Case two: Binary serialisation of complex, high-risk objects

Training AI models employing Python’s binary object serialisation can lead to patient data being embedded several layers deep in various object properties. A list containing objects was serialised, including a dictionary with AI models and their performance data. While the performance data was safe, a Support Vector Machine (SVM) included samples of the original training data in its support vectors, an acknowledged issue with SVM models.^25^ The training data had been Z-transformed, which could be reversed using population aggregates requested for egress.

**Figure 2.**
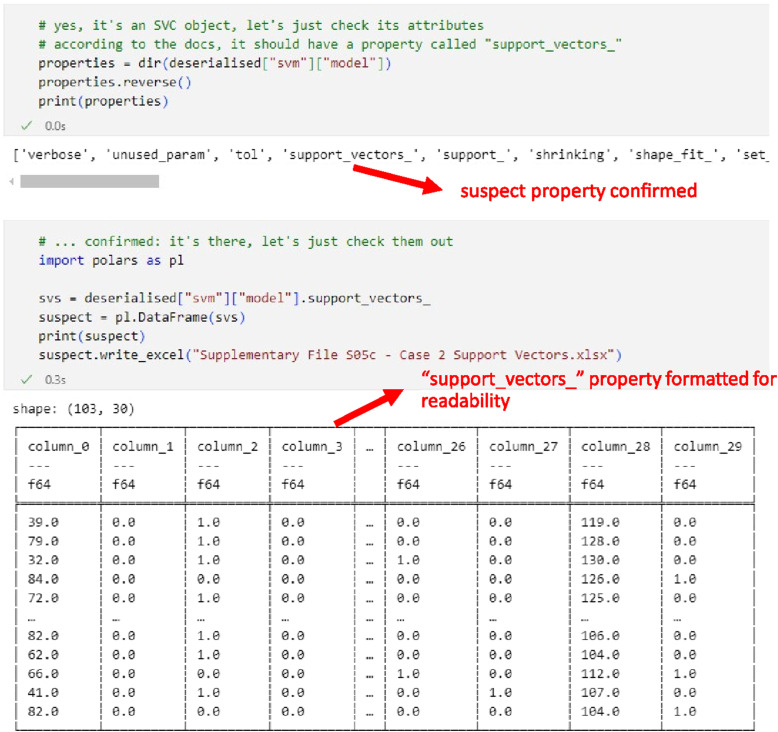
Case two as viewed in VS code. Screenshot of VS code showing fragment of Supplemental Code File S05b wherein the presence of support vectors was confirmed for a support vector machine classifier object embedded in Online Supplemental File S05a. Highlighted in red are the points where the suspect property’s presence in the object was confirmed, and a rendering of the property’s contents.

The SVM classifier was small in terms of kilobytes (kB) size but contained patient-level data in its support vectors. Online Supplemental File S05a contains a worked example of this case which includes synthetic patient-level data in a SVM binary object in a complex, multilayered binary-serialised object, while Online Supplementary File S05c contains the support vectors in tabular format, extracted from the SVM exported in Online Supplemental File S05b, which contains steps a technician might take to investigate the binary file, with the last step identifying the contents of the SVM in the screenshot of Figure 2 below.

The embedded data is identifiable with prior knowledge of potential safety risks associated with certain models, in this case the support vector machine. Here, the file size would probably be large but the SVM itself not necessarily so. Manual checking through multiple layers: list, dictionary, SVM, and support vectors, verifying contents by reversing the Z-transformation identified the data in question; see also Table 2 below for a full list of the properties of this object.

**Table 2.** Instance properties of a support vector machine classifier trained using the popular SciKit-Learn Python package (“SVC” class defined in the sklearn.svm namespace).

Property	Type
decision_function_shape	str
break_ties	bool
kernel	str
degree	int
gamma	str
coef0	float
tol	float
C	float
nu	float
epsilon	float
shrinking	bool
probability	bool
cache_size	int
class_weight	NoneType
verbose	bool
max_iter	int
random_state	NoneType
_sparse	bool
n_features_in_	int
class_weight_	numpy.ndarray
classes_	numpy.ndarray
_gamma	numpy.float64
support_	numpy.ndarray
**support_vectors_**	**numpy.ndarray**
_n_support	numpy.ndarray
dual_coef_	numpy.ndarray
intercept_	numpy.ndarray
_probA	numpy.ndarray
_probB	numpy.ndarray
fit_status_	int
_num_iter	numpy.ndarray
shape_fit_	tuple
_intercept_	numpy.ndarray
_dual_coef_	numpy.ndarray
n_iter_	numpy.ndarray

Highlighted in gray is the property that contained a large subset of the patient-level training data identified in Case three. Note also that in Python any one of the properties of a class object can be assigned at-will by external code, and so it is trivial to assign any arbitrary numpy.ndarray object (a data matrix of fixed type but arbitrary dimensions) to any of the relevant properties of a class that normally contains them – the name and normal function of the property are absolutely not reliable indicators as to the content of the property in any given instance when egressing data.

In hypothetical scenarios, object properties could be replaced with data that may be exfiltrated, resulting in unintentional disclosure. For instance, ndarray types in the Support Vector Classifier can be replaced with arbitrary data, bypassing detection unless directly examined.

### Learning

File size alone did not reliably indicate the presence of patient-level data, as the SVM model itself was small in terms of kilobytes, despite containing sensitive information within its support vectors. Additionally, the binary object serialisation technique employed in Python did not trigger any automated warnings or alerts regarding the presence of embedded patient data. It was only through thorough manual inspection and domain expertise that the potential risk was identified, particularly given the known issue of SVM models retaining samples of the training data. The ability to identify such data requires a strong understanding of both the AI models involved and potential data safety risks.

Binary serialisation, while convenient, introduces risks such as compatibility issues with library updates, security concerns (e.g., remote code execution vulnerabilities), and difficulties in detecting manipulated or exfiltrated data. The risks are not confined to Python or R but extend to any platform where text-based serialisation might be manipulated to evade normal detection procedures.

### Case three –Plain-text markup reports

This case’s main report was generated by the Quarto R library (identifiable only by tags in the report itself) and contained a mixture of JavaScript, JSON and HTML, which leveraged the base-64 encoding of binary data to embed arbitrary objects – Excel files in this case – as plain-text code in the html file which can later be decoded and opened by loading the html document in a web browser. The base-64 encoded text appears to anyone looking at it as a string of random numbers and letters sometimes suffixed with an “=” sign depending on the size of the object being encoded. For example, a UTF-8 encoded string containing the text “hello world” is represented as “aGVsbG8gd29ybGQ=” in base-64. Any binary data can be encoded as base-64, usually as a means to store the object as plain text, such as embedding it in an HTML document, as in this case. The objects in question were originally .xlsx files from Microsoft Excel, which are themselves zip-compressed XML files.^26^ Note here that decoding the base-64 string into its original binary form does not by itself reveal the file format or, therefore, its contents, which must be deduced by other means such as the magic number of the file signature (the first few bytes of the binary data the file contains), attempting to open it in a plain text editor, or, in this case, from the HTML markup into which the object was embedded which contained an attribute labelling the type of data encoded in the base-64 string.

The risk in this case includes the egress of aggregate numbers where low patient counts (sometimes equal to one) are unsuppressed. A third-party library lacking robust maintenance of its file outputs could embed data in HTML files as comments or as an unused variable in JavaScript, making detection challenging.

**Figure 3.**
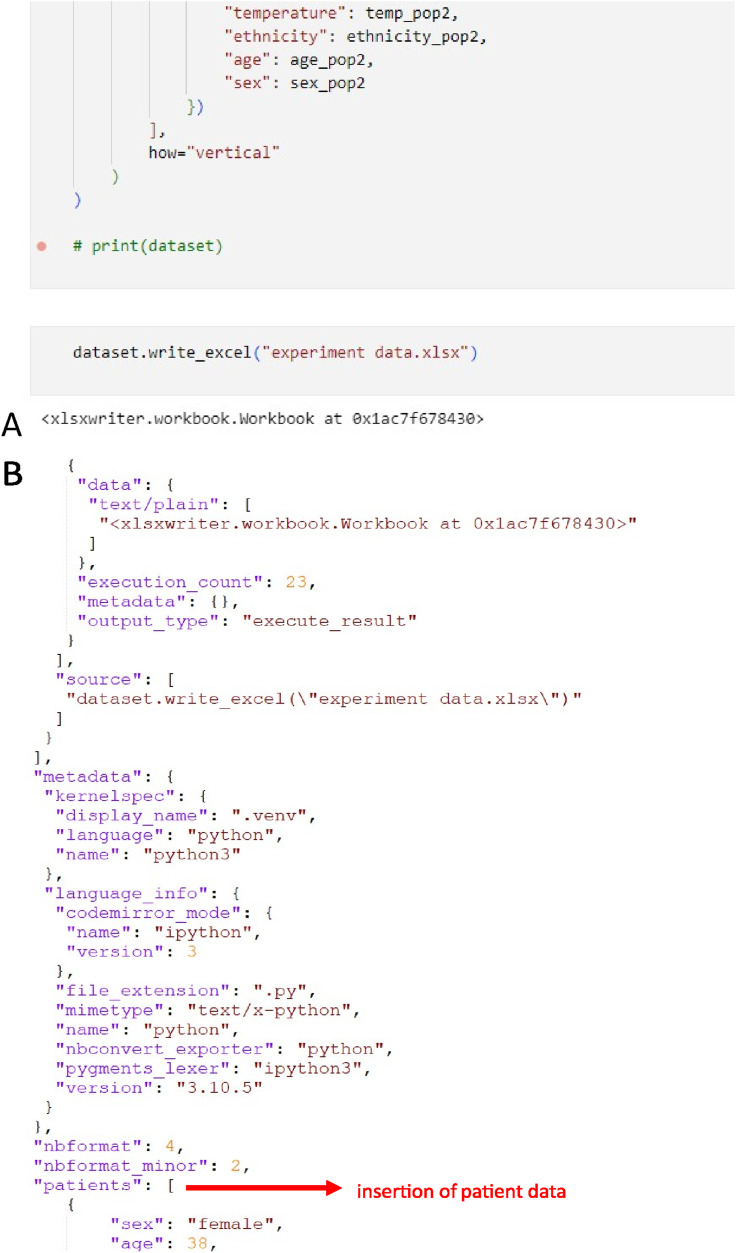
Patient data embedded in a Jupyter Notebook. Partial screenshots of the code markup file in Supplemental File S03. A shows a screenshot of the end of the file as rendered in VS Code at the location into which the synthetic patient data was inserted, and B shows the markup for the same screen area displayed in a plain text editor (Notepad++), marked in red is the site in which synthetic patient data has been inserted.

Online Supplemental File S03 contains a worked example which includes patient-level data embedded in a Jupyter Notebook file, a commonly used type of JSON-encoded markup document. Figure 3 provides a series of marked screen shots demonstrating the embedded synthetic data within the Jupyter notebook file.

In this instance the embedded data is detected by manual inspection of the markup document without rendering, in a plain text editor. A further complication in Case three is the embedding of file data in the markup document as Base-64 encoded text, which required specialised knowledge to identify and verify and would not be achievable by non-technical users.

### Learning

Once again, the file size did not serve as an immediate indicator of the presence of embedded patient-level data, as markup documents like HTML can vary in size depending on their content and verbosity. Similarly, the program did not issue any warnings about embedded data, as the use of Base-64 encoding to embed Excel files within the HTML file, a common practice, went undetected. It was through manual inspection using a plain-text editor that the embedded data was identified.

This case demonstrates that while tools can generate comprehensive, interactive reports using markup languages like HTML, they introduce significant challenges for verification, particularly when dealing with embedded data or Base-64 encoding, which requires specialised knowledge to detect and interpret.

There are risks associated with embedding patient-level data in images or metadata within these markup documents, as well as with third-party libraries that might not robustly manage file outputs, potentially allowing hidden data to be included without detection. To mitigate these risks, a more secure and efficient solution is to export simple, plain-text aggregate data with suppressed small numbers, facilitating safe reporting and formatting outside the SDE environment. This method minimises the risk of unintentional data egress and allows for continued access to formatted reports without exposing sensitive data. Table 3 provides a summary of the cases.

**Table 3.** Summary of all cases discussed herein, including the description, data involved, method of export, challenges faced, insights gained, guidelines, and researcher and TRE operator responsibilities for practitioners to mitigate similar risks.

Case	Description	Data involved	Format	Challenges	Insights	Guidelines	Researcher responsibilities	TRE operator responsibilities
Case one (High Risk)	Unintentional inclusion of performance data from AI models in binary-serialised files.	Individual level data on infections, patient demographics, diagnoses, comorbidities, lab results.	Binary object serialisation (Python’s pickle method).	Lack of awareness of permissible exports; complexity of verification process for pickled files.	Tools like object serialisation can easily include sensitive data; thorough manual inspection required for verification.	Educate researchers on permissible exports; implement rigorous verification processes for pickled files.	Avoid binary serialisation for egress (e.g., do not use .pkl for output); organise and document outputs clearly.	Provide explicit training on export-safe formats; reject high-risk formats when safer options exist; ensure manual or automated checks of any serialised objects.
Case two (Critical Risk)	Unintentional embedding of a Support Vector Machine with training data in a complex object structure that was binary-serialised.	AI models for clinical decision support, patient-level training data.	Binary object serialisation (Python’s pickle method).	Complexity of object structure; difficulty in identifying and verifying embedded data.	Complexity of object structure can conceal sensitive data; expertise of platform and platform-independent risks posed by different AI models required for thorough verification.	Develop expertise in platform used and knowledge of AI models; implement recursive inspection processes for complex objects.	Understand disclosure risks of certain ML models (e.g., SVC support vectors) and choose safer algorithms or anonymisation when exporting models.	Train staff to detect ML models that can store original data; enforce policy to decline export of high-risk models or require alternative representations.
Case three (High Risk)	Export of aggregated results as a complex and large set of markup language with embedded files.	Aggregate data without low-number suppression, arbitrary contents of embedded files.	HTML	Complexity of object structure, technical demands on verification.	Plain-text markup documents can easily be spiked by malicious users and require technicians with good expertise to verify their contents. Requires implicit trust in library creators that users cannot accidentally embed sensitive data.	Thoroughly inspect code and image metadata for sensitive data; ensure expertise in verification process, encourage use of plain-text aggregate data rather than formatted reports.	Provide outputs as plain-text aggregates with small-number suppression; avoid embedding external files in markup.	Require removal of all embedded files/metadata; perform raw-source inspection; supply automated tools to strip or flag hidden content.

To consolidate the insights from the three case studies, we developed a comparative framework that summarises the nature of the egress risks, their detectability, the potential for automation, and associated policy implications. Table 4 provides an integrated overview of how risks arise across different file types and analytical workflows.

**Table 4.** Summary of egress risks across the three case studies, including detectability, automation feasibility, and policy implication.

Dimension	Case 1: Binary serialisation of data	Case 2: Binary serialisation of complex ML objects	Case 3: Mark-upreports with embedded files
Primary egress vector	Embedded data matrices	Support vectors containing training data	Base-64 embedded spreadsheets/hidden content
Detectability	Moderate (visible after loading)	Low (deeply nested, requires expertise)	Low–Moderate (requires raw markup inspection)
Automation feasibility	Medium (pickle inspection possible)	Low (requires model-aware parsing)	Medium (metadata stripping + pattern detection)
Policy implication	Restrict pickle exports	Flag ML models as high-risk artefacts	Require plain-text outputs; suppress small counts

**Figure 4.**
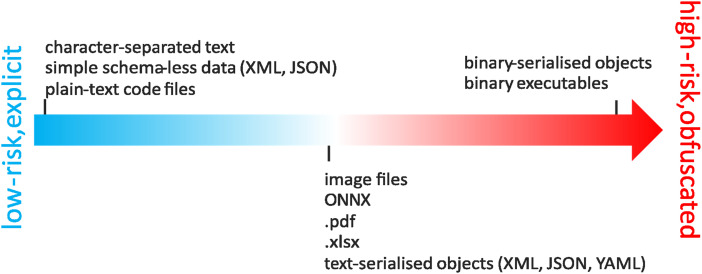
Spectrum of risk for files egressed from the PIONEER TRE, the scale of which depends on the type of file proposed for egress. Plain-text is fully human-readable, but depending on the way it is used can be relatively complex in nature, but always explicit. Non-human-readable formats, typically binary objects derived from arbitrary, user-defined types present the greatest risk.

In all cases presented, the hazard presented was potential exfiltration pathways, illustrating some examples where accidental data egress could occur, and the processes which can be used to mitigate this. Figure 4 provides a summary of the cases and the risks identified, as well as mitigating principles and practices.

## Discussion

In this study we demonstrate, using three worked examples, that individual-level patient data can be inadvertently egressed from TREs through common analytical outputs. The scenarios, binary serialisation of data, binary serialisation of complex machine-learning objects, and plain-text mark-up reports, showed that file size or format alone cannot reliably indicate risk and that automated detection is insufficient. Manual inspection and domain expertise remain critical for safeguarding data. These findings align with wider socio-technical models of secure system design, where multiple imperfect layers of oversight are required to reduce risk, rather than relying on any single safeguard.^15,16^

Although the worked examples were derived from operational experience within the PIONEER Trusted Research Environment, the mechanisms illustrated represent broader classes of artefact-level risk that may arise across Trusted Research Environments and Secure Data Environments more generally. Similar analytical workflows, programming libraries, and model artefacts are widely used across research infrastructures internationally. Consequently, the examples presented here should be interpreted as demonstrations of potential egress pathways that may occur in a variety of TRE implementations rather than vulnerabilities unique to a specific platform.

Comparison across the three worked examples highlights a spectrum of risk profiles shaped by file structure and analytical workflow. Binary serialisation of simple datasets (Case 1) presented moderate detectability, as embedded matrices became visible once objects were loaded, allowing semi-automated inspection approaches. In contrast, complex machine-learning artefacts such as support vector classifiers (Case 2) exhibited lower detectability because patient-level information was deeply nested within model properties, requiring specialist knowledge and manual inspection. Mark-up reports with embedded files (Case 3) occupied an intermediate position, although technically text-based, hidden encoded content reduced transparency and required inspection of raw source structures. These comparisons illustrate that automation alone is insufficient across all contexts; rather, risk mitigation depends on matching inspection strategies to artefact complexity. Automated tools may provide efficient triage for simpler outputs, while higher-risk artefacts require expert-driven review within governance frameworks.

The findings can be interpreted along an automation–manual inspection continuum. Automated tools provide scalable initial screening but remain limited in detecting context-dependent risks such as embedded patient-level information within complex objects. Manual inspection introduces expert judgment capable of identifying subtle or emerging risks but presents challenges related to scalability and consistency. Effective TRE governance therefore requires a hybrid approach combining automated triage with targeted expert review.

As the volume and complexity of analytical outputs produced within TREs continue to increase, the scalability of purely manual inspection processes becomes an important operational consideration. While expert review remains essential for identifying complex artefact-level risks, automated triage tools may help prioritise outputs that require deeper inspection. Hybrid approaches combining automated scanning with targeted human review may therefore provide a pragmatic balance between efficiency and assurance. Future work should evaluate and benchmark such automated detection tools across different analytical ecosystems to determine their effectiveness and operational feasibility within real TRE workflows.

Although our examples used synthetic datasets, the same mechanisms would apply to real patient data and potentially pose greater privacy risks because re-identification is possible. The worked examples highlight the importance of rigorous egress screening, suppression of small counts, and careful validation of serialised objects whenever real health data are analysed and exported.

Accessing sensitive data such as health data using TREs is becoming commonplace, supported by significant UK government investment.^2^ Operating standards for these environments have primarily focused on data access rather than output governance, reflecting a commonly held belief that code and model artefacts present low egress risk because any embedded patient-level data would be overtly visible through file name, type, or size. Our findings challenge this assumption. Similar to other secure environments, the governance of TREs involves balancing data-privacy protections with the needs of timely and efficient research and often relies on manual inspection, data minimisation strategies, and operational judgement.

The worked examples build on earlier theoretical concerns about inadvertent leakage of identifiable data from TREs.^27^ Our cases go further by offering concrete demonstrations of those risks. The purpose of these examples is threefold. First, to show that previously identified theoretical risks represent practical risks that Data Controllers must actively manage. Second, to provide specific illustrations of how individual-level data may be obfuscated within files or objects so that TRE personnel can develop processes to detect and remove embedded patient-level data. Third, to raise awareness among researchers developing statistical or machine-learning models that certain coding shortcuts can unintentionally embed patient-level data. Understanding these risks provides researchers with an opportunity to adopt safer practices. Jefferson et al.^13^ similarly proposed project-stage recommendations emphasising collaborative strategies for managing risk.

Within the PIONEER TRE screening prior to egress is performed manually by exploring research outputs and inspecting contents and metadata on a case-by-case basis. This covers a wide range of possible file formats, data structures, and content, but generally entails several common steps:

1. TREs should first assess whether an egress request is appropriate and permitted under existing policies and governance. If the risk or resource demands for checking are too high, the TRE may reject the request or work with the researcher to identify safer, lower risk alternatives.
2. Validation of file content as expected from file extensions, e.g. that a file with the extension .jpeg is, indeed a JPEG image.
3. Assessment of the risk of a file containing embedded data structures that may hold sensitive data, e.g. .csv file contents are readily verified, while complex binary structures are much harder to verify the safety of.
4. Inspection of file contents, which can range from simple visual inspection in the case of small text-based files such as .csv or code files, to recursive inspection of the properties of serialised objects.
5. Presentation and sign-off by senior personnel.

This mirrors practice in other secure environments such as in Australian law enforcement’s “Data Airlock” secure environment.^28^ Manual review, however, presents challenges regarding scalability, timeliness, and consistency. Automated tools can assist by flagging anomalies, such as unexpected file types, metadata discrepancies, or unusually large files, but they cannot determine whether embedded content is sensitive.^17,19,29,30^

Our findings reinforce the growing recognition that managing data egress from TREs requires more than conventional disclosure control focused on tabular outputs. The SDAP Handbook on Statistical Disclosure Control for Outputs^31^ provides practical recommendations for reviewing statistical outputs, but its emphasis is primarily on structured tabular data and visualisations. The cases presented here, especially the binary serialisation of machine-learning artefacts and embedding of data within mark-up documents, expand the risk landscape beyond what SDAP currently addresses.

The GRAIMATTER^13^ project highlighted the potential for trained ML models to contain personal data and therefore fall within the scope of data protection legislation. Our case studies operationalise this concern, showing how support vector machines and other models can retain or reconstruct training data. These empirical findings support the view that ML model objects should be treated as potential personal data during egress.

Recent UK projects such as SACRO^32^ and SATRE^33^ likewise prioritise secure analytics and output checking for ML and code-based outputs. Our findings are consistent with their recommendation for layered controls, including pre-approval of file types, enhanced staff training, and risk-based triage. They also complement ongoing work by NHS England and the Department of Health and Social Care to establish an SDE accreditation process aligned with ISO 27001. While these initiatives strengthen baseline standards, our results suggest that accreditation schemes should explicitly consider serialisation controls, model-artefact inspection, and the risks demonstrated here.

Taken together, these comparisons show that our proposed guidelines; restricting binary serialisation, promoting plain-text outputs, and enforcing robust manual and automated inspection, extend existing national and international guidance. Moreover, they highlight a key policy implication: TREs should explicitly assess whether an egress request is appropriate before output checking begins and decline requests where the risk is disproportionate to the research need.

Although manual inspection remains indispensable, full manual review is not scalable for modern analytical workflows. A practical approach is a triaged, defence-in-depth system in which automated tools perform initial screening and expert reviewers focus on high-risk outputs.^15–17^ This layered approach aligns with GDPR principles of data minimisation and privacy-by-design and mirrors international frameworks such as HIPAA and the EU Data Governance Act.

The findings are directly relevant to Data Controllers, TRE/SDE operators, and researchers. They underscore the need for clear egress policies, ongoing training, and investment in automated tools that can surface embedded structures requiring human evaluation. Integrating these tools within a layered governance framework will support consistent, risk-proportionate decision-making across TREs.

These findings should therefore be interpreted as illustrative demonstrations of risk pathways rather than empirical measurements of disclosure likelihood. The value of the cases lies in highlighting how commonly used analytical workflows may create unexpected governance challenges.

Building on the case studies, we propose the following recommendations for TRE practice:

• Policy Gatekeeping: Before any technical review, assess whether the egress request is permissible under TRE policy and data-use agreements.
• Mandatory Researcher Training: Require training on risks of binary serialisation, embedded metadata, and model inversion before granting access.
• Tiered Egress Checks: Combine automated triage (e.g., file-type and size scanning, metadata stripping) with expert manual inspection for high-risk files.
• Approved Output Formats: Restrict export to plain-text or other human-readable formats unless a strong justification is provided and risk assessment completed.
• Ongoing Auditing and Feedback: Implement continuous audit trails and provide structured feedback to researchers to improve future compliance.

These measures align with GDPR principles of data minimisation and accountability and complement NHS Digital’s SDE accreditation framework. While existing NHS Digital SDE guidance and SACRO/SATRE frameworks establish baseline operational standards, the contribution of this work lies in demonstrating concrete technical failure modes that these policies do not yet explicitly cover, such as model-object inversion risks and hidden Base-64 artefacts. Our recommendations extend existing guidance by proposing dynamic operationalisation, including semi-automated risk scoring of output types, model-aware artefact scanning, and embedding code-level safeguards (for example disabling pickle egress, enforcing plain-text outputs). These measures move beyond policy reiteration to provide actionable, technically grounded enhancements for TRE operations.

This study has several limitations. First, although synthetic data ensured ethical compliance, it cannot fully replicate the complexity, heterogeneity, and edge-case behaviours of real clinical datasets. The mechanisms of data embedding and leakage demonstrated here would operate identically on real data, but the diversity and granularity of real health records may increase the difficulty of detecting hidden patient-level information. Repeating this analysis with real-world data, with appropriate ethical approvals, would be needed to demonstrate this. Second, only three representative cases were examined. Other analytical ecosystems (for example SAS, Stata, SQL stored procedures, deep-learning artefacts) may pose additional risks not captured here. Third, the risk assessment relied on expert manual inspection, which is inherently subjective and may vary between TREs. Fourth, manual review is resource-intensive and challenges GDPR principles of data minimisation if reviewers must interrogate more information than strictly necessary. Finally, although we outline mitigation strategies, these have not yet been operationalised or stress-tested at scale; future work should evaluate how automated triage, model-artefact scanning, and risk-scoring approaches perform in real TRE workflows.

The examples provided are not intended as representative of synthetic-data methods but instead illustrate how individual-level data might be embedded in exported code or artefacts. While synthetic-data techniques could be improved, doing so would not alter the lessons learned. Moreover, training generative models on patient data or deriving statistics for certain synthesis methods would itself require a lawful basis and appropriate governance controls.

## Conclusion

The contribution of this work lies in demonstrating mechanisms of potential data egress and identifying governance considerations, rather than establishing generalisable risk estimates across TRE infrastructures. The findings indicate that commonly used analytical outputs; including binary serialised data objects, complex machine-learning models, and plain text mark-up reports; can conceal patient-level information in ways that are not detectable through routine automated egress checks. File size or format alone is not a reliable indicator of risk. Manual inspection by knowledgeable personnel remains essential until more robust automated detection tools are developed. The findings highlight the need for TREs and SDEs to incorporate explicit egress risk assessment alongside access controls to prevent inadvertent disclosure of patient data.

## Supplemental material

Supplemental material - Identifying patient-level data risks in Trusted Research Environments: Worked examples with synthetic data Supplemental material for Identifying patient-level data risks in Trusted Research Environments: Worked examples with synthetic data by M. Baragilly, A. Topham, S. Gallier and E. Sapey in Digital Health.

## Data Availability

To facilitate knowledge in this area, the synthetic data, analytical code and a data dictionary defining each field will be available to others through application to PIONEER via the corresponding author.
