# Supplementary material for: Identifying patient-level data risks in trusted research environments: Worked examples with synthetic data
Source: Digit Health. 2026 Jun 23;12:20552076261440981. doi: 10.1177/20552076261440981 (PMC13305797; doi:10.1177/20552076261440981)
Supplement: Supplemental material - Identifying patient-level data risks in Trusted Research Environments: Worked examples with synthetic data [file sj-pdf-1-dhj-10.1177_20552076261440981.pdf]

Main repository is held at:

https://github.com/pioneer-alexander-topham/Patient-Level-Egress-Risk-TREs

Python version is 3.13.1

For library versions, see `requirements.txt`

## Supplementary File S01

`SupplementaryFiles/Supplementary File S01 - Random Data Generator.ipynb`

## Supplementary File S01a - ONS Ethnicities.xlsx

`SupplementaryFiles/Supplementary File S01a - ONS Ethnicities.xlsx`

## Supplementary File S02a - Transformed Data.xlsx

`SupplementaryFiles/Supplementary File S02a - Transformed Data.xlsx`

## Supplementary File S02b - Data Feature Modelling.ipynb

`SupplementaryFiles/Supplementary File S02b - Data Feature Modelling.ipynb`

## Supplementary File S02c - Feature-Modelled Data.xlsx

`SupplementaryFiles/Supplementary File S02c - Feature-Modelled Data.xlsx`

## Supplementary File S02d - Model Training.ipynb

`SupplementaryFiles/Supplementary File S02d - Model Training.ipynb`

## Supplementary File S03 - Markup Document with Hidden Data.ipynb

On GitHub, open this file and select the “Raw” view to see the file markup in plain text. Alternativey, on a local machine, open the file in your plain text editor of choice (NotePad++, Notepad, Kate, Nano etc) Scroll to the bottom and you will see the last entries of the “hidden” data, which are invisible when rendered by an application (like GitHub in non-raw mode, or Visual Studio Code) that processes the markup.

`SupplementaryFiles/Supplementary File S03 - Markup Document with Hidden Data.ipynb`

## Supplementary File S04 - Case 1 Outputs.pkl

`SupplementaryFiles/Supplementary File S04 - Case 1 Outputs.pkl`

## Supplementary File S05a - Case 2 Outputs.pkl

`SupplementaryFiles/Supplementary File S05a - Case 2 Outputs.pkl`

## Supplementary File S05b - Case 2 investigation.ipynb

`SupplementaryFiles/Supplementary File S05b - Case 2 investigation.ipynb`

## Supplementary File S05c - Case 2 Support Vectors.xlsx

`SupplementaryFiles/Supplementary File S05c - Case 2 Support Vectors.xlsx`
